# Learning tools used to translate resilience in healthcare into practice: a rapid scoping review

**DOI:** 10.1186/s12913-023-09922-6

**Published:** 2023-08-23

**Authors:** Cecilie Haraldseid-Driftland, Heidi Dombestein, Anh Hai Le, Stephen Billett, Siri Wiig

**Affiliations:** 1 Centre for Resilience in Healthcare, Faculty of Health Sciences, University of Stavanger, N-4036 Stavanger, Norway; 2 School of Education and Professional Studies, Griffith University, Mount Gravatt, QLD 4122 Australia

**Keywords:** Resilience, Healthcare, Quality, Collaborative learning, Systems perspective, Learning tools

## Abstract

**Background:**

Historically, efforts to improved healthcare provisions have focussed on learning from and understanding what went wrong during adverse events. More recently, however, there has been a growing interest in seeking to improve healthcare quality through promoting and strengthening resilience in healthcare, in light of the range of changes and challenges to which healthcare providers are subjected. So far, several approaches for strengthening resilience performance have been suggested, such as reflection and simulation. However, there is a lack of studies that appraise the range of existing learning tools, the purposes for which they are designed, and the types of learning activities they comprise. The aim of this rapid scoping review is to identify the characteristics of currently available learning tools designed to translate organizational resilience into healthcare practice.

**Methods:**

A rapid scoping review approach was used to identify, collect, and synthesise information describing the characteristics of currently available learning tools designed to translate organizational resilience into healthcare practice. EMBASE and Medline Ovid were searched in May 2022 for articles published between 2012 and 2022.

**Results:**

The review identified six different learning tools such as serious games and checklists to guide reflection, targeting different stakeholders, in various healthcare settings. The tools, typically, promoted self-reflection either individually or collaboratively in groups. Evaluations of these tools found them to be useful and supportive of resilience; however, what constitutes resilience was often difficult to discern, particularly the organizational aspect. It became evident from these studies that careful planning and support were needed for their successful implementation.

**Conclusions:**

The tools that are available for review are based on guidelines, checklists, or serious games, all of which offer to prompt either self-reflection or group reflections related to different forms of adaptations that are being performed. In this paper, we propose that more guided reflections mirroring the complexity of resilience in healthcare, along with an interprofessional collaborative and guided approach, are needed for these tools to be enacted effectively to realise change in practice. Future studies also need to explore how tools are perceived, used, and understood in multi-site, multi-level studies with a range of different participants.

**Supplementary Information:**

The online version contains supplementary material available at 10.1186/s12913-023-09922-6.

## Background

Providing safe, high-quality care is the principal goal for healthcare systems worldwide [1]. Historically, efforts to improved healthcare provision have focussed on learning from and understanding what went wrong during adverse events [2]. Yet, more recently, there has been a growing interest in trying to improve healthcare quality from a resilience in healthcare perspective, and in understanding how high-quality care is provided despite the range of changes and challenges to which healthcare providers are subjected during service provision [3–5].

Importantly, there is a broad consensus amongst researchers that resilience of healthcare systems needs to be strengthened [6]. Recent studies within the field of resilience in healthcare have explored the underlying potentials for resilience [7], described adaptive capacities for resilience [8], defined the boundaries and concept of resilience [9], and sought to understand what contributes to resilience in practice by describing what occurs in ordinary work processes and how work as done differs from work as imagined [10, 11]. Consequently, there is now a growing consensus that the concept of resilience in healthcare entails the capacity to adapt to challenges and changes at different system levels in order to provide high-quality care [9]. It is also acknowledged that adaptive capacities contribute to generating resilient healthcare systems [8] and that these adaptive capacities usually require an element of collaborative learning and working, as the complexities of changes and challenges can rarely be addressed by individuals alone or by single healthcare disciplinary knowledge [12]. Resilience in healthcare is a systems perspective. This is of importance since it places the responsibility for providing high-quality care on the system and the organisation, rather than on the individual. For a system to operate in a resilient manner, it needs to provide the individuals within it with the equipment and resources to enable resilient performance. As such, resilient healthcare is dependent on organisational learning, where the organisation continuously assimilates new knowledge and improves and adapts its systems’ routines, rules, and performances based on both existing and newly assimilated knowledge [13]. Organisations’ ability to strengthen resilient performance is therefore dependent on intentional efforts to promote continuous and collaborative learning to improve resilient performance of the system [7, 9]. To aid such processes, healthcare organisations, leaders, and staff need practical tools to drive continuous learning processes and to facilitate the understanding of what constitutes resilience in healthcare and understandings of how resilience in healthcare can be strengthened [12].

Resilience in healthcare builds on the concept of resilience engineering that stems from other fields such as cognitive psychology and safety science [14] and has been widely used in other sectors such as aviation [15] and nuclear power [16]. As a result, tools for operationalising resilience engineering have been developed, such as serious games for industrial safety focusing on developing early warning indicators [17] or ‘Five steps to resilient decision making’ aiming at develop insight into resilience strategies [18]. There are also tools related to resilience engineering that focus on the aspect of proactive learning, such as proactive assessment of organisational and workplace factors (PAOWF) that provides organisations with an overview of how conditions change [19] and the proactive risk monitoring tool for organisational learning in healthcare (PRIMO) that focuses on potential failures [20]. In addition, there are tools focusing on resilience within disaster planning [21, 22] and others for studying and understanding resilience performance in healthcare, such as the resilience analysis grid (RAG), the functional resonance analysis methodology (FRAM), and the concepts for applying resilience engineering (CARE) model [14, 23, 24]. Nevertheless, there has been a growing call for interventions that operationalise and translate organisational resilience into practice [25–28]. To date, two different approaches for strengthening resilience performance have been proposed broadly: reflection, where current practices and ideas are thought about and discussed, and simulation, where real life events are imitated, practiced, and rehearsed [29–31]. However, there is a lack of studies that systematically identify and appraise such learning tools, for which purpose they are designed, and what type of learning activities they entail. This article begins to address this gap.

### Aim and research questions

The aim of this rapid scoping review is to identify and appraise the characteristics of currently available learning tools designed to translate organisational resilience into healthcare practice.

The research questions are:

1. What learning tools are available to translate organisational resilience into healthcare practice?
2. What are the characteristics of these tools?

In referring to these characteristics, we focus on type of tool, aim, pedagogical activities, pedagogical approach, and possible outcomes from implementing the tool.

## Methods

A rapid scoping review approach was used to identify, collect, and synthesise information describing the characteristics of currently available learning tools designed to translate organisational resilience into healthcare practice. The scoping review follows the methodological framework developed by Arksey and O’Malley [32], with improvements by Peters et al. [33]. A rapid review technique described by Tricco et al. [7, 34] was adopted as it allows for rapid collection of evidence to map a field of study. The method is compliant with the PRISMA-ScR reporting guideline and checklist [35], and is used to inform this review. Five stages of activities were enacted in the review: (a) identifying the research question, (b) identifying relevant studies, (c) study selection, (d) charting the data, and (e) collating, summarising, and reporting the results [32].

### Identifying the research question

In the last decade, resilience in healthcare as a research field has expanded rapidly. Due to the novelty of the field, a majority of the work has been focusing on theory building and defining the boundaries and concept of resilience in healthcare [7–10]. However, with the field developing, several studies have called for interventions helping to operationalise resilience in healthcare [25, 27, 28]. Before developing new tools and interventions it is thus timely and appropriate to conduct a fresh scoping review over the existing literature to enable future studies to build on and learn from this knowledge. A rapid review approach was adopted since this methodology is particularly helpful in capturing emerging tools and practices in a resource-efficient way [36].

### Identifying relevant studies

The search terms were selected to align with the population-concept-context (PCC) components that entail defining a search string for each component and subsequently combining them into the final search string [33, 37]. EMBASE and Medline Ovid were searched in May 2022. According to Tricco et al. [34], only two databases are required for a rapid scoping review, which allows for a rapid collection of evidence to map a field of study. Medline Ovid was chosen to capture health service related studies, while EMBASE was chosen to capture interdisciplinary studies. EMBASE also indexes health system research that is not indexed in Medline; as such, the two databases complement each other. The search was limited to include peer reviewed articles published in English between 2012 and 2022. The design of the search string was developed with support from a specialised librarian; the final search was conducted by one author (AL). Table 1 outlines the literature search and its categorising into the participant, concept and context (PCC) framework. To ensure that all relevant articles were identified, reference scanning of included articles and a manual search in Google Scholar and relevant journals (such as BMC Health Services Research and Applied Ergonomics) were conducted by two authors (CHD and HD).

**Table 1:** Search string EMBASE and Medline Ovid

Search no	Query	Result
#1 **Participant**	**'healthcare personnel'**/exp OR **'healthcare personnel'** OR worker* OR 'healthcare worker*' OR **'personnel'**/exp OR **personnel** OR leader* OR manager*	2,344,910
#2 **Concept**	'learning tool*' OR tool* OR **'serious games'** OR **reflection** OR **'reflexive practice'** OR **'collaborative learning'**	1,244,191
#3 **Concept**	resilien* OR **'resilience engineering'** OR safety*ii OR **'safety 2'** OR **'adaptive capacity'** OR adapt* OR 'organizational resilien*'	962,671
#4 **Context**	**healthcare** OR **'health care'**	2,601,316
#5	**#1** AND **#2** AND **#3** AND **#4**	4,126
#6	**#5** AND (**2012**:py OR **2013**:py OR **2014**:py OR **2015**:py OR **2016**:py OR **2017**:py OR **2018**:py OR **2019**:py OR **2020**:py OR **2021**:py OR **2022**:py)	3,315
#7	**#5** AND (**2012**:py OR **2013**:py OR **2014**:py OR **2015**:py OR **2016**:py OR **2017**:py OR **2018**:py OR **2019**:py OR **2020**:py OR **2021**:py OR **2022**:py) AND **'article'**/it	1,887
#8	**#5** AND (**2012**:py OR **2013**:py OR **2014**:py OR **2015**:py OR **2016**:py OR **2017**:py OR **2018**:py OR **2019**:py OR **2020**:py OR **2021**:py OR **2022**:py) AND **'article'**/it AND (**'case study'**/de OR **'evidence-based practice'**/de OR **'intervention study'**/de OR **'qualitative research'**/de OR **'systematic review'**/de)	279

### Study selection

**Fig. 1:**
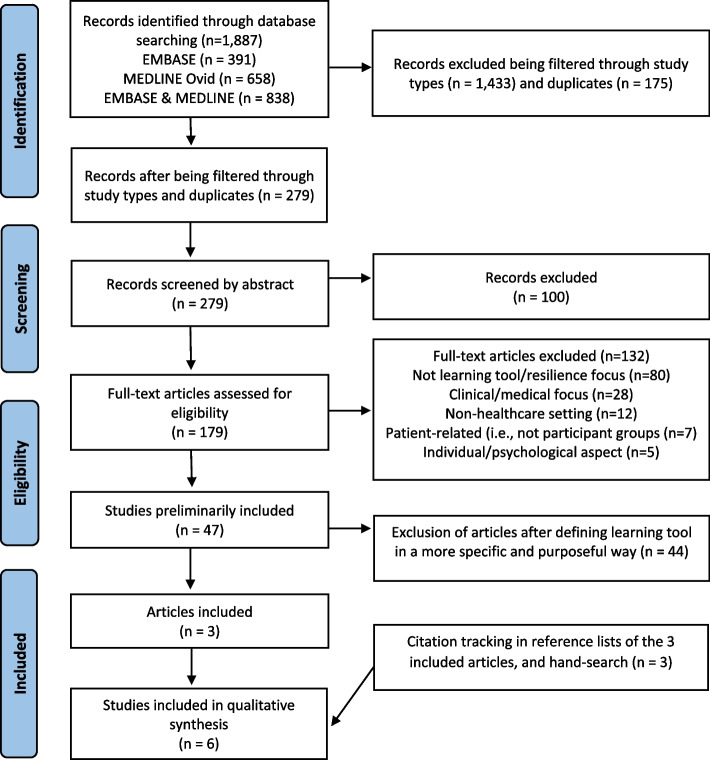
PRISMA flow diagram

Based on keywords in the research questions and familiarity with the literature, inclusion and exclusion criteria were developed (see Table 2). The inclusion criteria used in our rapid scoping review related to type of participant groups; type of setting; descriptions of learning tool; descriptions of organisational resilience; and type of study. One author (AL) applied the inclusion and exclusion criteria to all the records to determine their relevance. In line with the rapid review methodology [34], two authors (CHD and HD) independently checked 10% of the excluded records to ensure that relevant records were not excluded on incorrect premises. No discrepancies among the excluded studies were detected. Checking the initial included full text articles indicated that the search figure and inclusion criteria had identified a large number of studies that described the concept of learning tool in a broad way. These studies did not have the potential to address the research questions, as they either: lacked descriptions of the learning tool used, only described tools that enabled researchers to study work processes rather than operationalising and strengthening resilience concepts, or that the tools were based on a different concept of resilience stemming from other fields such as cognitive psychology or disaster planning which address other processes such as individual behaviour rather than collaborative efforts. The concept of learning tool was, therefore, defined in a more specific and purposeful way that included a means to support practitioners to practically translate resilience into practice, in order to identify articles that could help meet the research questions of operationalising resilience into healthcare practice. Two authors (CHD and HD) further included or excluded articles based on this revised definition (see Fig. 1).

**Table 2:** Inclusion and exclusion criteria

Inclusion criteria	Exclusion criteria
Healthcare settings	Non-healthcare settings i.e., work-life or education
Describe learning tool and/or use of tools that aim to translate resilience in healthcare into practice by strengthening resilience in healthcare features such as adaptations, adaptive capacity, safety II, learning, anticipation, monitoring, responding into healthcare settings	Describing individual or psychological resilience (describing personal/psychological/individual/worker/professional resilience) Individual/psychological aspect Clinical/medical focus Not learning/resilience focus Resilience engineering
Must include description of tool and characteristics of tool, designed for use by workers, personnel, and/or leaders in healthcare	Without description of tool or tool designed for use by others than personnel and leaders in healthcare such as researchers/patients/students etc
Empirical studies and systematic reviews Evidence-based practice, intervention studies, and case studies	Commentary, theoretical articles, editorials, opinion papers
Qualitative, quantitative, or mixed-methods designs	Studies without methods description
Peer reviewed articles reporting academic output	Not reporting academic output and book chapters, books, conference proceedings, and grey literature
Articles in English	Language other than English
Published between 2012 and 2022	Published before 2012

Consistent with the scoping review methodology, no quality appraisal was conducted to exclude articles based on that assessment. As scoping reviews seek to develop a comprehensive overview of the field rather than a quantitative or qualitative synthesis of data, there is no requirement to undertake methodological appraisal/risk of bias assessment of the sources to be included [32].

### Charting and summarising the data and reporting the results

Two authors (CHD and HD) extracted key items of information obtained from each primary article into a data charting form [32]. A draft of the charting form was developed to allow efficient data coding. The form was left open to allow editing and additional unforeseen data during the analyses, which permitted the process to be iterative. The data extraction process focused on the (a) aim of the studies, (b) setting and participants, (c) type and descriptions of the tool, (d) pedagogical approach and activities, and (e) possible outcomes from implementing the tool (please see Appendix 1 ‘Data charting form’ for details of the extracted data). To chart the data further, authors SB and AL created a complementary descriptive summary of the results, addressing the objectives and research questions stated in the introductory section of this review, consistent with the Preferred Reporting Items for Systematic Reviews and Meta-Analyses Extension for Scoping Reviews reporting checklist (PRISMA- ScR) [35].

## Results

The search and descriptive analysis results are now presented and discussed below.

### Search results

The searches revealed 1,887 articles from the databases, of which 1,608 were filtered through study types using exclusion criteria (e.g., commentary, theoretical, editorial, studies without method description), thereby reducing the number of articles to 279. After reviewing their titles and abstracts, 100 articles were excluded because they did not meet the inclusion criteria regarding settings, language, or focus of the study (i.e., not learning/resilience, non-learning tool, clinical/medical focus, individual/psychological aspect). The findings reported in those studies failed to satisfy the study’s objectives in terms of reporting resilience learning tool in healthcare settings, leaving 179 full-text articles to be assessed for eligibility. After full-text reviews, 176 additional studies were excluded due to the following: inconclusive findings about resilience learning tools (e.g., no resolution to enhance resilience or discussion of resilience as a healthcare concept), not being conducted in healthcare contexts, and not empirical studies, reviews of perspectives or discussing resilience as an individual/psychological aspect or tools for researchers not practitioners. Additional reference scanning of the three included articles and a manual search in Google Scholar and relevant journals resulted in three additionally included articles. The complete process, then, resulted in six articles being identified for qualitative descriptive analysis. Workflow of the identification, screening, eligibility, and inclusion of the studies in the rapid scoping review according to the PRISMA flow diagram [34, 37] can be found in Fig. 1.

### Description of tools, pedagogical approach, and evaluation

In the analysis, each tool was assessed according to the type and descriptions of the learning tool, pedagogical approach and activities, and outcomes and evaluations of the tool (see Table 3). Pedagogical approach entails how the learning tool was designed to be used, either alone (individually) or in pairs/or groups (collectively). The pedagogical approach is of particular interest since collaborative learning is an important aspect of resilient healthcare since healthcare is provided as a shared effort between a range of stakeholders [12].The analysis across the tools provided insight into the patterns, needs, and characteristics needed to better succeed with a learning tool to translate resilience into practice and into what further studies need to emphasise.

**Table 3:** Summary of characteristics of the included studies

Reference	Description of tool		Pedagogical approach		Tool evaluation
	***Scenario-based***	***Checklist***	***Individually***	***Collaboratively***	
Bartman et al. (2021) [38]		√	√	√	Testing
Bentley et al. (2021) [39]		√		√	Survey (*n* = 10)
Jackson et al. (2020) [40]	√		√		Survey (*n* = 107)
Hegde et al. (2020) [41]		√	√		Discussion
Hermelin et al. (2020) [42]		√	√	√	Survey (*n* = 9)
Wahl et al. (2022) [43]		√		√	Survey (151 respondents)

The descriptive analysis of the results indicated that there are learning tools targeting different stakeholders (e.g., clinical and professional staff at different levels, residents, practitioners, and policymakers) in various healthcare settings (e.g., hospitals, healthcare centres, simulation centres). As presented in Table 3, all six articles identified a tool involving self-reflection either individually or collaboratively in groups. Whilst one was designed using scenarios formulated in a game (i.e., serious game), the other five involved checklists to guide reflections on resilience capacities. Surveys were used to evaluate the implementations of the four tools, while research group discussion and pilot testing was used to evaluate the other two.

In detail, Jackson et al. [40] reported on the serious computer-based game ‘Resilience Challenge’ in a hospital setting, aiming to communicate principles of resilient healthcare to clinicians and to prompt reflection on practice related to safety and organisational resilience. In this tool (i.e., the game), the players follow the patient through their hospital journey (e.g., emergency department, orthopaedic ward, x-ray, medical ward, etc.) and adopt multiple healthcare roles throughout the game (e.g., registered nurse, doctor, nurse administrator). In each scenario, the players face different dilemmas in making decisions to advance the game. The players are given feedback for every choice made to prompt their reflection. This tool was evaluated through an online survey with 107 participants (i.e., players). The design (i.e., flow images and sound) was well received: the players reported that it helped them reflect on their own practice related to safety issues, and how their actions influenced other parts of the system. Concerns were raised regarding the fact that the game had ‘right’ answers, and the lack of understanding of organisational resilience for most participants.

Also in a hospital setting, Bartman et al. [38] developed a checklist with short statements to be used as mental prompts to predict, respond to, and promote learning. Examples of these statements include ‘pause to predict’, ‘gather information’, or ‘discuss with at least two team members’. This tool was designed to improve clinical frontline staff’s situational awareness, anticipation, responding, and learning, and to adapt to patients’ conditions. Initial testing of a prototype found an elevation in formal and more guided proactive safety huddles to effectively address unusual and potential harmful situations.

Contextualised for anaesthetists, Hegde et al. [41] developed a tool in the form of an online questionnaire (i.e., resilience engineering tool to improve patient safety [RETIPS] for anaesthesia residents [RETIPS-AnRes]) in which respondents are asked to write a narrative where an adaptation occurred and to specify what worked well, which challenges and concerns triggered a particular response, and what resources were used. This tool was used to elicit narratives of adaptations that have contributed to effectiveness in care delivery. Based on discussions in the article, the researchers concluded that the tool supported the concept of learning how things go well in everyday work through helping the participants to reflect. However, the article also discussed that the tool is limited in its focus on individual learning and lacked an emphasis on and basis in realising change in organisational practice.

Situated in a simulation centre, Bentley et al. [39] developed a debriefing checklist with exemplary questions for how to guide the debrief of clinicians from different disciplines and simulations into resilience capacities. The tool aims to facilitate inclusion of Safety-II analysis into debriefings through questions related to variability, adaptability, flexibility, and workarounds. The tool implementation was evaluated through a survey with 10 participants; all reported that this tool would add value to their debriefings and that it was understandable and easy to use.

To improve patient safety through learning from everyday work, Wahl et al. [43] developed the Green Line reflection tool as a short assessment. The reflection sessions were administered as 5- to 10-min huddles with all available staff (i.e., nurses, nurse assistants, managers, and doctors). Reflections and discussions were based on open-ended questions such as ‘How have we succeeded today?’ and follow-up questions such as ‘How did you manage that?’ and ‘Can you describe more?’. Ideas for improvement were noted during each huddle to promote collaborative learning. An evaluation survey was conducted with 151 evaluative responses for the tool. Participants reported difficulty in introducing reflections based on learning from what goes well, as this was an unfamiliar approach. Also identified was the need for careful planning and support from managers’ knowledge of the underpinning theory of this approach to staff learning and organisational enhancements.

In an article broadly addressed to policymakers, managers, and practitioners, Hermelin et al. [42] reported on guidelines to enhance organisational resilience in crisis management and capability development. These guidelines comprised 13 cards of generic descriptions of capabilities of resilience, called the DARWIN resilience management guidelines (DRMG; DARWIN, 2019). In addition to reflections (i.e., both group and individual approaches), the activities outlined in the tool comprised lectures, workshops, and table-top simulations in which different scenarios and ways to be prepared and adaptable to diverse situations were discussed and appraised. There were also suggestions for suitable interventions or actions for all kinds and levels of staff capabilities and a set of triggering questions to guide the self-reflections. The tool implementation was evaluated through a survey with only nine participants; positive feedback was received, indicating the content was deemed relevant and interesting for the participants.

### Patterns across learning tools

These six tools offer a variety of approaches rather than a variety of tools. This means that all tools offered some form of scenarios and examples as the basis for individual and collective consideration and reflection and, in four of the six tools, collaborative processes of engagement in addition to individual engagement. What is perhaps missing in this mix is some form of guidance that assists the engagement and effective translation into practice and the contributions of these tools.

In all, from this review of relevant literature, three key elements emerge. Firstly, a process is needed that goes beyond individual reflections and accommodates collective and, ideally, interprofessional interactions as processes of collaboration. This implies that the potential learning experiences cannot be limited to individual learning and development alone, but also require engagement through and outcomes associated with collaborative engagement and learning. Secondly, the tools’ scenarios, vignettes, or narratives used as the key focuses for that engagement include elements that address workplace or work practice requirements: that is, more than prompting individual learning, they are positioned to consider and realise changes in work practices. Thirdly, given the complexity of the task of translating these means into resilience and practice and the interprofessional engagements required, some guidance is likely to be needed to facilitate and realise the full learning potential of these tools. Hence, the means through which these tools can be enacted may well need to include a guide, facilitator, or advocate of some kind to facilitate and support the reflections towards different and interconnected levels and layers of resilience.

## Discussion

This rapid scoping review found that only a limited number of learning tools exist that can translate resilience into healthcare practice. Six tools were identified: one game and five guides/checklists. In the following, we discuss the findings and suggest patterns and needs for future research.

### Reflection: most used approach

All six tools included some form of reflection: through self-reflection [40, 41], group reflections [39, 43], or a combination of both [38, 42]. Reflection is a commonly known pedagogical approach which aims to stimulate learning through interpreting and making sense of an experience, and using that experience to guide decision-making and actions [44]. Several other studies have recommended reflections as the preferred method in terms of trying to enhance or understand the concept of resilience in healthcare. Wiig et al. [30] argued that critical reflection helps to articulate tacit knowledge which could bridge experience, knowledge, and action. Lyng et al. [8] argued that tools that facilitate reflection can support and strengthen resilience performance, perhaps most effectively when those reflections are centred and focused on actual instances, as exemplified here. Resilience in healthcare is a complex concept in which a range of different adaptations are enacted and performed to provide high-quality patient care; hence, collaborative approaches to reflective practice are required. This provides the opportunity to view each situation as unique, and to take into account a range of aspects rather than applying a standardised solution [45]. As such, this might explain why reflection as a pedagogical approach is a good fit within efforts to advance resilience.

Resilience in healthcare has a systems dimension, meaning that it is not only individuals but rather the system that needs to enact resilience [9, 46, 47], hence collaborative engagements are important. While individual or self-reflection can be useful for understanding the concept of resilience, it is limited in its reach. On the one hand, a strong individual focus, when the aim is to promote organisational resilience, could be problematic. On the other hand, the link between individual and organisational resilience is somewhat unclear, as it is the individuals within the organisation who make up the teams and the organisation and are, as such, a potentially important contribution to the organisational phenomenon. A more holistic view that includes individuals as well as a systems-based approach might therefore be needed for strengthening resilience in healthcare; future research is, therefore, suggested to investigate this relation in more detail.

### Difficulties grasping the concept

Three of the articles [40, 41, 43] reported that participants had difficulties in understanding and grasping the organisational aspect of resilience. This is, perhaps, not surprising, given the novelty of adjusting to a new approach of focusing on what goes well in a situation rather than focusing on adverse events, which has been the orthodox approach to enhancing patient safety [3, 48]. Learning new skills and approaches, especially when they differ markedly from previous learnt skills, is demanding [49]. In most of the studies, reflection was related to discussing adaptations that were made and why. Enhancing systems resilience requires understanding of how individual, group, and systems adaptations interplay with and across participants, and how different levels and stakeholders within the healthcare systems contribute to systems resilience [8]. All of this points to the importance of having a collaborative element to the processes through which these tools are enacted. Given the complex and multi-partied nature of resilience, the reason participants struggled to understand the concept of resilience might be that the processes used to promote reflections failed to address this complexity. Instead, there is the risk that participants linked reflections only to the act of individual adaptations rather than incorporating and discussing the multiple layers of understanding how different stakeholders contribute to organisational resilience. To better accommodate this complexity, reflection activities might benefit from a more guided and collaborative approach related to different aspects of resilience that press them to consider system-level implications and practices, as suggested by Lyng et al. [8].

### Interprofessional collaboration, cross sectional and cross levels: a solution to the problem?

Within the six included articles, four had a group approach, all of which included some sort of interprofessional collaborative element. Interprofessional collaboration, where multiple professions work together to achieve shared goals [50], is a commonly utilised and long-standing approach in organisations, institutions, and professions to achieve better outcomes than through single professions alone [51]. Such collaborations are also known for creating opportunities to learn and expand knowledge beyond orthodox ways of thinking [52], providing opportunities to tackle new, large, and complex problems through gathering and sharing information amongst different groups [53]. This was seen in Wahl et al. [43], who reported that the safety huddles helped to create common values and cohesion between staff members who were commonly working in different areas, which is perceived as an important aspect of resilience in healthcare [8]. Interprofessional group reflections could, thereby, enhance the potential to strengthen resilience. However, several of the included studies [40, 41, 43] reported difficulties in grasping the concept of resilience and, in particular, the organisational aspect. Interprofessional group reflections might, therefore, not be sufficient. Inclusion of both different professions and levels might be needed to facilitate the understanding of the organisational aspects, potentially strengthening resilience performance through identifying and discussing diverse aspects of practice and their implications at a range of levels within the organisation [54]. While individual reflective efforts can be useful to support capacity building and the conditions to enhance resilience performance, tools for resilience in healthcare should include a cross-level, collaborative, and interprofessional approach. This is necessary to facilitate understanding and accommodation of the organisational aspect of resilience in healthcare and how resilience performance is created through a collaborative effort across stakeholders and levels [55, 56].

### Lack of studies and small study samples

This review identified a lack of tools for operationalising and translating resilience in healthcare into practice. In addition, the evaluations of the tools were characterised by using small sample sizes, mostly restricted to the hospital setting. There was also limited reporting on the outcomes of the tools in the included studies. Those that were reported were limited to focus on the design, user friendliness, and value and frequency of tool usage. The small scale of these samples, as well as the focus of the evaluation, questions the veracity of the findings and prompts the need for more comprehensive and interprofessional approaches to the evaluation of such tools. The complexity of the resilience in healthcare concept also warrants a more comprehensive evaluation design, one that focuses not only on value and frequency of use, but that also evaluates the whole implementation process and outcomes.

The lack of studies also indicates an underdeveloped field and strengthens the objectives of this paper, as it underscores the need for a summary of characteristics of the existing tools to prompt further growth in this field of interest. Although the tools identified are deemed both interesting and useful, the studies also report difficulties in ‘getting the message across’ to the participants. As mentioned above, resilience in healthcare is a complex, multilevel phenomenon which researchers and practitioners have struggled to define, explain, and operationalise [9]. This might be the reason that several of the studies identified through the initial searches reported on tools to help researchers understand, explain, and map resilience characteristics [14, 23, 24]. The novelty of the field and the complexity of enacting these interventions might also explain why the studies included in this paper are mostly single-site and small-scale studies with a limited number of participants. The field of resilience in healthcare draws on the resilience concept stemming from other sectors such as societal safety, engineering, and societal ecology and psychology [14, 57]. Some of these sectors offer a relatively extensive range of tools [17–19] that could provide valuable contributions to the field of resilience in healthcare. Future studies with cross-site, multi-level, and interprofessional settings, drawing on the previous work both within resilience in healthcare and similar work in other resilience sectors, are therefore needed.

### Limitations and strengths

The rapid scoping study approach does not seek to assess quality of evidence and consequently cannot determine whether particular studies provide robust or generalisable findings [32]. Conducting a quality appraisal of included articles, even if this is not demanded according to the methodology, could have strengthened our study. On the other hand, the rapid scoping review methodology allows information from a broad range of peer-reviewed articles, that used diverse designs and methods, to be included and synthesised. The limited number of studies found and discussed in this paper might be related to the novelty of the area, but it is also noted that several contributions to the field are published within books [58–61] instead of in peer reviewed articles. Choosing to include book chapters, conference proceedings, and grey literature might have broadened the findings; however, the peer review process for such publications is sometimes lighter and to strengthen the validity of the study it was chosen to include only papers that had gone through the strict peer review process of journal article publications.

In this study it was chosen to exclude studies with a pure resilience engineering background. The identified papers in this category were deemed unfit for inclusion since they covered areas such as disaster planning or loss of power and infrastructure [21, 62] and as such did not describe tools that enabled translation of organisational resilience into healthcare practice. However, future studies should explore resilience tools stemming from other sectors such as engineering, oil and gas, nuclear power, and how these can be of value for the healthcare sector.

Finally, the studies included in this rapid scoping review had different approaches and were used in different contexts. Future studies could benefit from exploring how the different settings influence the use of such tools.

## Conclusion

Through this review, we have identified six learning tools designed to translate organisational resilience into healthcare practice and the characteristics of these tools in terms of their aim, pedagogical approach and activities, and possible outcomes from implementing the tools. The review found a limited range of learning tools to translate organisational resilience into healthcare practice. The tools that are available are based on guidelines, checklists, or serious games, all of which offer to prompt either self-reflection or group reflections related to different forms of adaptations that are performed.

Based on the findings, a more guided, collaborative, and interprofessional approach to such reflective processes is warranted. In this way, they may come to mirror the complexity of resilience in healthcare. Future studies need to explore how tools are perceived, used, and understood in cross-site, multi-level studies with a range of participants. Moreover, the current evaluation of tools available to translate resilience into practice is limited by small samples and limited appraisals across varying healthcare contexts. Larger studies with mixed methods designs, including cross level and cross context (nursing homes, homecare, hospitals, mental health, GP, low- and high-income countries), are warranted and recommended. Due to the methodological limitations of rapid scoping reviews, a comprehensive systematic review is recommended when conducting further knowledge synthesis.

### Supplementary Information

**Additional file 1.**

## Data Availability

All articles that were used as basis for data are available online.

## Abbreviations

SHARE: Centre for Resilience in Healthcare
RiH: Resilience in Healthcare
