# Supplementary material for: Learning tools used to translate resilience in healthcare into practice: a rapid scoping review
Source: BMC Health Serv Res. 2023 Aug 23;23:890. doi: 10.1186/s12913-023-09922-6 (PMC10463810; doi:10.1186/s12913-023-09922-6)
Supplement: Supplementary file 1 — Additional file 1. [file 12913_2023_9922_MOESM1_ESM.docx]

| **Nr.** | **Article** | **Aim of the study** | **Setting and participants** | **Type and description of the tool** | **Pedagogical approach and activities** | **Possible outcomes from implementing the tool** |
| --- | --- | --- | --- | --- | --- | --- |
| 1 | Jackson J, Iacovides J, Duncan M, et al. Operationalizing resilient healthcare concepts through a serious video game for clinicians. *Applied Ergonomics* 2020;87:103112. doi: <https://doi.org/10.1016/j.apergo.2020.103112> | Communicate principles from resilient healthcare to clinicians and prompt reflection on practice related to safety and organizational resilience. | Hospitals,  Healthcare professionals | Serious game. The player follows the patient through his hospital journey (emergency department, orthopaedic ward, x-ray, medical ward ect.) The player takes on multiple healthcare roles throughout the game (RN, doctor, nurse administrator). The player faces different dilemmas for each scenario and must make decisions to advance the game. | Self-reflection. Resolve dilemmas in clinical practice. Each dilemma is based on a misalignment where the player must adapt to the changing circumstances, none of the options are ideal, but one of the options are more acceptable for all parties involved. Only one of the options advances the game. The player is given feedback for every choice made to prompt reflection. Individual approach through participation in a computer-based game. | Evaluated through an online survey with 12 questions (N=107). Design (flow images and sound) was well received, the players found that it helped them reflect over own practice related to safety issues, and how their actions influenced other parts of the system. Some disagreement concerning the fact that the game had ‘right’ answers. Lack of understanding of organizational resilience for most participants. |
| 2 | Bartman T, Merandi J, Maa T, et al. Developing Tools to Enhance the Adaptive Capacity (Safety II) of Health Care Providers at a Children's Hospital. *Jt Comm J Qual Patient Saf* 2021;47(8):526-32. <https://doi.org/10.1016/j.jcjq.2021.03.006> | Improve staff’s situational awareness, anticipation, responding and learning and to adapt to patients conditions | Hospitals,  Clinical frontline staff | Checklist with short statements to be used as mental devices to predict, respond and learn. Examples from the tool: Pause to predict, gather information, discuss with at least two team members). | Self-reflection and group discussions. Individual approach with collaborative elements | Initial testing of prototype found an elevation in formal and more guided proactive safety huddles to tackle unusual and potential harmful situations. |
| 3 | Hegde S, Hettinger AZ, Fairbanks RJ, et al. Qualitative findings from a pilot stage implementation of a novel organizational learning tool toward operationalizing the Safety-II paradigm in health care. *Applied Ergonomics* 2020;82:102913. doi: <https://doi.org/10.1016/j.apergo.2019.102913> | Elicit narratives of adaptions that have contributed to effectiveness in care delivery | Hospital  Anaesthesia residents | List of questions  RETIPS-AnRes.  Online questionnaire where the respondent is asked to write a narrative where an adaption took place, and specify what went right, which challenges and concerns that triggered the response and what resources were used in the situation. | Self-reflection with an individual approach | Based on discussions: The researchers conclude that the tool supported the concept of learning how things go well in everyday work though helping the participants reflect. Focus on individual learning and a lack of organizational learning. |
| 4 | Bentley SK, McNamara S, Meguerdichian M, et al. Debrief it all: a tool for inclusion of Safety-II. *Advances in Simulation* 2021;6(1):9.  <https://doi.org/10.1186/s41077-021-00163-3> | Facilitate inclusion of safety II analysis into debriefings | Simulation center, clinicians from different disciplines and simulationists | Debriefing checklist with exemplary questions for how to guide the debrief into resilience capacities | Group discussion/reflection.  Group based approach | Survey (n=10). All participants believed this tool would add value to their debriefings, it was understandable and easy to use. |
| 5 | Hermelin J, Bengtsson K, Woltjer R, et al. Operationalising resilience for disaster medicine practitioners: capability development through training, simulation and reflection. *Cogn Tech Work* 2020;22(3):667-83.  <https://doi.org/10.1007/s10111-019-00587-y> | Enhance organizational resilience in crisis management/ capability development | Policy makers, managers, practitioners, | Resilience guidelines called Capability cards, (DRMG), Generic descriptions of capabilities of resilience (13). Suggestions for suitable interventions or actions for all capabilities and a set of triggering questions to guide self-reflection. | Lectures, workshops, table-top simulation, group reflection, Self-reflection.  Both group and individual aspects. | (Survey n=9). The content was deemed relevant and interesting for the participants. |
| 6 | Wahl K, Stenmarker M, Ros A. Experience of learning from everyday work in daily safety huddles—a multi-method study. *BMC Health Services Research* 2022;22(1):1101.  <https://doi.org/10.1186/s12913-022-08462-9> | Improve patient safety through learning from everyday work | Healthcare workers (nurses, nurse assistants, managers and doctors) | The Green Line, 5–10-minute huddle between all available staff. Reflection and discussion based on open ended questions such as ‘How have we succeeded today?’ Follow up questions such as ‘How did you manage that?’ and ‘Can your describe more?’. Ideas for improvement were noted during each huddle to promote learning. | Group reflection/discussions. Group based approach | Survey (151 respondents). Difficult to introduce reflections based on learning from what goes well. Need careful planning and support form managers knowing the underpinning theory. |

**Additional file 1:** Appendix 1 Data charting form
